# Deep Single-Cell Transcriptomic Profiling of Bovine Milk Somatic Cells Revealed Expression of Stem Cell Related Transcription Factors

**DOI:** 10.3390/genes17040365

**Published:** 2026-03-24

**Authors:** Mateja Dolinar, Peter Dovč, Minja Zorc

**Affiliations:** Biotechnical Faculty, University of Ljubljana, Jamnikarjeva 101, 1000 Ljubljana, Slovenia; mateja.dolinar@bf.uni-lj.si (M.D.); peter.dovc@bf.uni-lj.si (P.D.)

**Keywords:** single-cell RNA sequencing, bovine milk, milk somatic cells, mammary gland, lactation, transcriptome, cattle, Holstein Friesian cows

## Abstract

**Background/Objectives**: Milk somatic cells reflect the cellular composition and functional state of the lactating mammary gland and represent a valuable, non-invasive source for transcriptomic studies. Single-cell RNA sequencing (scRNA-seq) enables cell-type-resolved analysis of bovine milk; however, sequencing depth strongly influences the detection of lowly expressed genes and the resolution of transcriptional cell states. The aim of this study was to further characterise the single-cell transcriptome of bovine milk somatic cells, with particular emphasis on high-resolution gene expression profiling and cellular heterogeneity. **Methods**: Milk somatic cells were isolated from two healthy Holstein Friesian cows in mid-lactation and profiled using a droplet-based scRNA-seq platform. Newly generated high-depth datasets were integrated with two previously published bovine milk scRNA-seq datasets using an identical bioinformatics pipeline. Data integration, clustering and cell-type annotation were performed using the Seurat framework, and transcription factor expression was evaluated across datasets with different sequencing depths. **Results**: Single-cell transcriptomic analysis revealed a diverse cellular landscape in bovine milk, comprising epithelial, progenitor, and immune cell populations. Unsupervised clustering identified 21 transcriptionally distinct clusters, including multiple CD8^+^ T-cell subpopulations, monocytes, neutrophils, mast cells, and B cells, as well as luminal epithelial and luminal progenitor cells. While overall cell-type composition was comparable across datasets, deeply sequenced samples exhibited higher transcriptomic complexity and enabled refined resolution of immune and epithelial subpopulations. Deeper sequencing facilitated the detection of low-abundance transcription factors that were not observed in lower-depth datasets. Among these, *NANOG* was detected exclusively in deeply sequenced samples, suggesting the presence of rare transcriptional states associated with cellular plasticity. **Conclusions**: This study expands the single-cell transcriptomic landscape of bovine milk somatic cells and demonstrates the importance of sequencing depth for resolving functional cellular heterogeneity. The results highlight milk as a powerful, non-invasive source for investigating mammary gland biology and cellular plasticity during lactation.

## 1. Introduction

Single-cell RNA sequencing (scRNA-seq) has become a central methodology for dissecting transcriptional heterogeneity in complex biological systems, enabling the investigation of gene expression variability at the level of individual cells rather than averaged populations [1,2]. By capturing cell-specific transcriptional profiles, scRNA-seq has revealed functional cell states, regulatory programmes, and dynamic transcriptional responses that remain inaccessible using bulk RNA sequencing approaches.

Lactation biology is particularly suitable for single-cell transcriptomic analyses due to the functional complexity and dynamic nature of the mammary gland. The mammary gland undergoes extensive structural and transcriptional remodeling throughout pregnancy, lactation, and involution, driven by coordinated gene expression programmes within epithelial and immune cell populations [3,4]. During lactation, mammary epithelial cells exhibit exceptionally high metabolic and secretory activity, while immune cells contribute to immune surveillance and tissue protection, making the mammary gland an informative system for studying transcriptional regulation at cellular resolution.

Milk provides a unique and non-invasive biological material for investigating mammary gland function. Milk somatic cells originate from exfoliated mammary epithelial cells and infiltrating immune cells and retain transcriptional signatures reflective of ongoing physiological processes within the gland [5]. Because milk can be collected repeatedly without disrupting tissue integrity, milk-derived cells enable longitudinal investigations of mammary gland activity across lactation stages and environmental conditions [6]. This non-invasive nature distinguishes milk from mammary tissue biopsies and has motivated its increasing use in transcriptomic studies.

Early transcriptomic analyses of milk somatic cells relied on microarray-based approaches and bulk RNA sequencing, which identified coordinated expression of genes involved in milk protein synthesis, lipid metabolism, immune response, and tissue remodeling during lactation [7,8,9]. While these studies provided valuable insights into lactation-associated gene regulation, bulk approaches inherently obscure cell-type-specific transcriptional programmes by averaging signals across heterogeneous cell populations. This limitation is particularly pronounced in milk, where epithelial, immune, and progenitor-like cells coexist in varying proportions.

The introduction of scRNA-seq enabled a shift from population-level to cell-level transcriptomic analyses, allowing direct investigation of cellular heterogeneity within the mammary gland. Single-cell studies of mammary tissue in humans and model organisms revealed transcriptionally distinct epithelial subpopulations, including luminal progenitor cells, differentiated secretory cells, and basal-like populations, as well as dynamic immune cell compartments [10,11,12,13]. These studies demonstrated that functional specialization within the mammary gland is often driven by transcriptional states rather than discrete cell identities.

Subsequent application of scRNA-seq to milk-derived cells further expanded mammary gland research by demonstrating that exfoliated cells in milk retain biologically meaningful transcriptional profiles. Single-cell analyses of human milk identified distinct epithelial and immune cell populations and revealed dynamic transcriptional changes associated with lactation stage and maternal factors, supporting the use of milk as a biologically informative source for single-cell studies [14,15].

Becker et al. (2021) [16] provided single-cell datasets of freshly isolated bovine milk cells, identifying epithelial and immune cell populations. Zorc et al. (2024) [17] characterised the single-cell transcriptome of bovine milk somatic cells during mid-lactation, revealing epithelial subpopulations, immune cell types, and genes contributing to transcriptional variability within milk-producing cells. Together, these studies positioned milk-derived scRNA-seq as a robust and biologically informative approach for studying bovine lactation at single-cell resolution. Ren et al. (2025) [18] applied scRNA-seq to milk cells from high- and low-lactation Holstein cows, generating a single-cell atlas of bovine milk cells. Through sub-clustering and cell–cell interaction analyses, this study identified epithelial and immune subpopulations associated with lactation performance and highlighted transcriptional pathways related to milk synthesis, immune signalling, and intercellular communication. These findings reinforce the concept that functional differences in lactation may be driven by transcriptional states within established cell populations rather than by differences in overall cell composition.

An important consideration in scRNA-seq experimental design is the balance between cell number and transcriptomic resolution. While profiling large numbers of cells increases statistical power for detecting rare populations and estimating cell-type proportions, enhanced transcript recovery per cell enables more detailed characterisation of gene expression programmes within defined cell types [19,20]. Sequencing depth strongly affects the detection of lowly expressed genes, including transcription factors, signalling molecules, metabolic regulators, and stress-response genes that may be critical for defining functional cell states but are often under-represented in sparsely sequenced cells [21].

In the context of lactation biology, deeper transcriptome coverage is particularly relevant for highly specialized secretory epithelial cells present in milk. In these cells, functional differences related to milk synthesis, metabolic activity, or immune interactions may be reflected by quantitative variation in gene expression rather than discrete cell-type boundaries. Enhanced transcript detection can facilitate the identification of pathways involved in milk protein synthesis, lipid metabolism, energy utilization, and cellular stress responses, as well as intra-population heterogeneity among epithelial and immune cells. Similarly, improved resolution of immune cell transcriptomes may provide insights into immune surveillance mechanisms and inflammatory processes influencing mammary gland health and milk quality [22].

Taken together, these observations highlight the value of complementary single-cell transcriptomic strategies that emphasize detailed gene expression profiling at single-cell resolution. Expanding and refining scRNA-seq datasets of bovine milk somatic cells contributes to a more nuanced understanding of mammary gland function during lactation and provides a foundation for future studies aimed at linking transcriptional states with phenotypic traits relevant to dairy production. The aim of this study was to generate and analyze high-depth scRNA-seq profiles of bovine milk somatic cells and, by reprocessing previously published datasets using an identical pipeline, to evaluate how sequencing depth affects the resolution of cellular heterogeneity and detection of low-abundance regulatory transcripts.

## 2. Materials and Methods

### 2.1. Animals and Milk Sample Collection

Milk samples were collected from two healthy Holstein Friesian cows in the late first lactation (both 265 days in milk). Two weeks before sampling the Cow3 had 16,000 and the Cow4, 99,000 milk somatic cells per mL. Animals showed no clinical signs of mastitis at the time of sampling. Milk was collected under standard farm conditions during the post-milking phase at the morning milking. For each cow, milk from all four quarters was pooled to generate a representative sample. Samples were immediately transported to the laboratory on ice and processed upon arrival. No invasive procedures were performed.

### 2.2. Isolation of Milk Somatic Cells

Upon arrival at the laboratory, the milk samples were processed immediately. Each milk sample was divided into three 50 mL tubes and centrifuged at 2000× *g* for 20 min at 4 °C. After defatting, the cream layer and supernatant were removed. Remaining cell pellets were washed in 35 mL of cold phosphate-buffered saline (PBS) and centrifuged to remove residual milk components. After washing, cells were resuspended in PBS containing bovine serum albumin (BSA) and filtered through 40 µm cell strainer to remove cell aggregates. The resuspended cells from the same cow were combined. Prior to library preparation cell concentration and viability were assessed using the Luna fluorescence cell counting method.

### 2.3. Single-Cell RNA Sequencing Library Preparation and Sequencing

Single-cell RNA sequencing libraries were prepared using the 10x Genomics (Pleasanton, CA, USA) Chromium Single Cell 3′ Gene Expression platform, following the manufacturer’s instructions. Briefly, single-cell suspensions were loaded onto the Chromium Controller to generate single-cell gel bead-in-emulsions (GEMs). Reverse transcription and cDNA amplification were performed according to the standard 10x Genomics workflow. Sequencing libraries were constructed using the Chromium Single Cell 3′ reagents. Prepared libraries were sequenced on an Illumina sequencing platform (San Diego, CA, USA) using paired-end reads. Sequencing depth differed between samples, resulting in variation in reads per cell between datasets.

### 2.4. Data Processing and Quality Control

Raw sequencing data were processed using Cell Ranger (10x Genomics) version 10.0.0. Reads were aligned to the bovine reference genome (ARS-UCD2.0). Quality control of single-cell RNA-sequencing data was performed in R (version 4.5.0) using the Seurat package (version 5.4.0) [23]. Cells were filtered using lower and upper thresholds for gene and unique molecular identifiers (UMI) counts. Cells were filtered using dataset-specific lower and upper thresholds for detected genes and unique molecular identifier (UMI) counts, determined from the empirical distributions (1st–99.5th percentiles) with minimums of 200 detected genes and 500 UMIs per cell. In addition, cells were filtered using fixed cutoffs for mitochondrial (≤20%), ribosomal (≤60%), and hemoglobin (≤5%) transcript proportions. Putative doublets were identified using scDblFinder (version 1.14.0) [24], implemented through the SingleCellExperiment framework (version 1.26.0) [25]. Doublet scores were computed based on neighborhood density in reduced expression space, and cells classified as doublets were excluded. Normalization and variance stabilization were performed separately for each library using SCTransform (v2), implemented in Seurat.

Two bovine milk somatic cell scRNA-seq datasets [17], available in the European Nucleotide Archive under accession number PRJEB73560, were reprocessed together with the newly generated datasets using the same reference genome (ARS-UCD2.0) and bioinformatic pipeline. These datasets correspond to samples collected at 75 and 93 days in milk and are referred to as Cow1 and Cow2.

### 2.5. Data Integration and Clustering Analysis

After quality control and normalization, datasets were integrated using the Seurat framework, which applies an anchor-based approach. Integration was carried out on SCTransform-normalized. Following integration, dimensionality reduction was performed using principal component analysis (PCA), and the first 30 principal components were used for downstream graph construction and UMAP visualization. Unsupervised clustering was subsequently applied using a graph-based community detection algorithm implemented in Seurat with a clustering resolution of 0.6.

### 2.6. Cluster Annotation and Cell-Type Identification

Cluster-specific marker genes were identified using the FindAllMarkers function in Seurat. Marker detection was performed by comparing cells within each cluster to all remaining cells in the integrated dataset, using a minimum expression threshold of 25% of cells per cluster and a log_2_ fold-change threshold of 0.25.

Cell-type annotation was performed based on canonical marker genes and cluster-specific marker genes identified in this study, in conjunction with a previously published bovine milk single-cell transcriptomic dataset [17]. Marker gene patterns were compared with known immune and epithelial cell markers, as well as with reference signatures reported in earlier studies of bovine milk somatic cells, to assign biologically meaningful cell-type labels to each cluster. Final cluster annotations were manually curated to ensure consistency with established cell identities and previously reported nomenclature.

### 2.7. Transcription Factor Analysis

Transcription factors (TF) were defined using a curated bovine transcription factor list obtained from AnimalTFDB [26]. TF expression was assessed in the integrated dataset using normalized expression values and summarized across samples and/or clusters. Average TF expression was computed per sample and visualized for the most abundant TFs.

To evaluate the impact of sequencing depth on TF detection, TF profiles were compared between deeply sequenced datasets (Cow3–Cow4) and lower-depth datasets (Cow1–Cow2), and TFs with low overall abundance were inspected for depth-dependent detectability.

## 3. Results

### 3.1. Overview of Single-Cell RNA Sequencing Datasets

Single-cell RNA sequencing was performed on milk somatic cells isolated from two healthy Holstein Friesian cows in mid-lactation (150 and 163 days in milk, respectively). In the Cow3 dataset, 263 high-confidence cell-associated barcodes were identified, with deep sequencing resulting in a mean depth of approximately 1.28 million reads per cell. This was reflected in high per-cell complexity, with a median of 1115 detected genes and 4638 UMIs per cell, and a sequencing saturation of 94.2%.

The Cow4 dataset comprised 1386 detected cells with a lower mean sequencing depth of approximately 286,841 reads per cell. Accordingly, median gene and UMI counts per cell were lower (382 genes and 970 UMIs), while sequencing saturation remained comparably high (94.8%).

To enable direct comparison, two previously published bovine milk somatic cell scRNA-seq datasets were reprocessed using the same reference genome assembly and an identical bioinformatic pipeline. These datasets, referred to as Cow1 and Cow2, correspond to samples collected at 75 and 93 days in milk (DIM), respectively. They contained substantially higher numbers of cells (10,073 and 17,781) but exhibited lower per-cell sequencing depth and complexity compared with the newly generated datasets (Table 1).

### 3.2. Quality Control of scRNA-Seq Datasets

Quality control was performed to remove low-quality cells, technical artifacts, and potential doublets prior to downstream analyses. Cells were filtered based on gene and UMI counts as well as the proportion of mitochondrial, ribosomal, and hemoglobin transcripts. The number of cells retained after quality filtering and doublet removal for each dataset is summarized in Table 2.

After quality filtering and doublet removal, 240 cells were retained in the Cow3 dataset and 992 cells in the Cow4 dataset. For the previously published datasets, 8921 cells (Cow1) and 15,906 cells (Cow2) passed all quality control steps.

The median proportion of mitochondrial transcripts remained low across all datasets, ranging from 0.17% to 0.82%, indicating good RNA integrity and minimal cellular stress. Median numbers of detected genes and UMI counts reflected the expected differences in sequencing depth, with higher complexity observed in deeply sequenced samples. Doublet rates were low and consistent across datasets, and doublets were removed prior to downstream analyses.

### 3.3. Data Integration and Clustering

**Figure 1:**
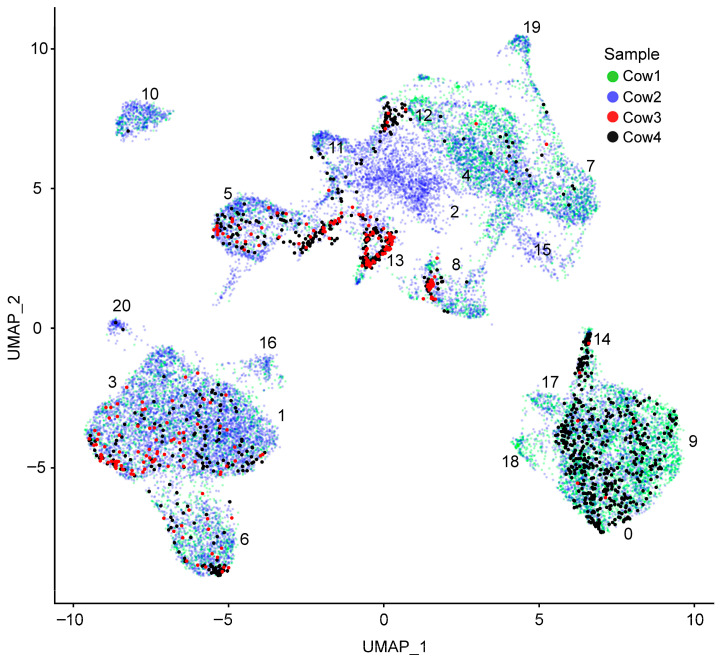
UMAP of integrated scRNA-seq dataset colored by biological sample. Numbers indicate cluster identifiers (clusters 0–20) as defined by unsupervised clustering.

Following quality control and normalization, all four datasets were integrated using the anchor-based integration framework. Uniform manifold approximation and projection (UMAP) of the integrated dataset revealed mixing of cells derived from different samples (Figure 1). Cells originating from the same sample did not form any distinct sample-specific clusters (no sample-specific segregation was observed). The datasets originating from samples with a lower number of cells are represented as a subset within clusters. This uneven representation reflects biological and sampling differences. Unsupervised graph-based clustering of the integrated dataset identified 21 clusters.

### 3.4. Identification of Major Cell Populations in Bovine Milk

**Figure 2:**
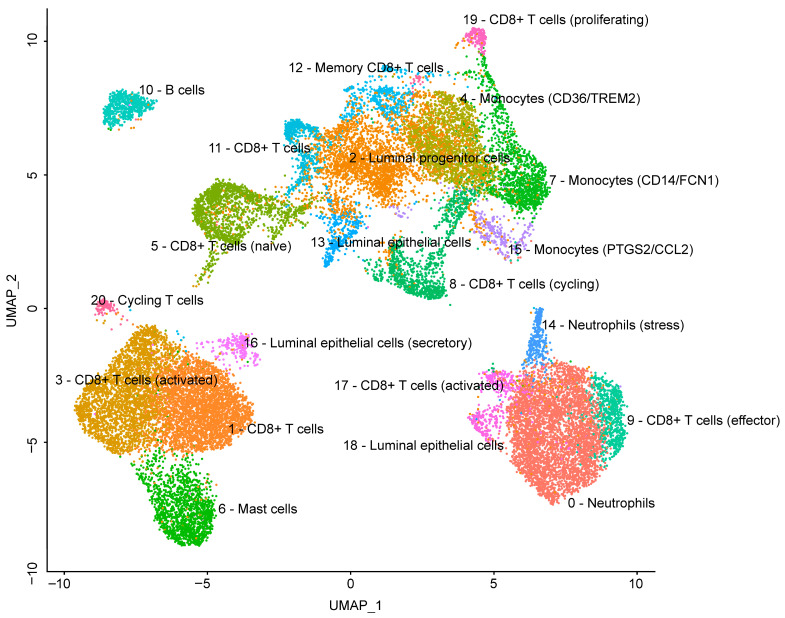
UMAP of integrated scRNA-seq dataset with cell-type annotations. Clusters are annotated as follows: 0—Neutrophils, 1—CD8^+^ T cells, 2—Luminal progenitor cells, 3—CD8^+^ T cells (activated), 4—Monocytes (*CD36/TREM2*), 5—CD8^+^ T cells (naive), 6—Mast cells, 7—Monocytes (*CD14/FCN1*), 8—CD8^+^ T cells (cycling), 9—CD8^+^ T cells (effector), 10—B cells, 11—CD8^+^ T cells, 12—Memory CD8^+^ T cells, 13—Luminal epithelial cells, 14—Neutrophils (stress-associated), 15—Monocytes (*PTGS2/CCL2*), 16—Luminal epithelial cells (secretory), 17—CD8^+^ T cells (activated), 18—Luminal epithelial cells, 19—CD8^+^ T cells (proliferating) and 20—Cycling T cells.

Unsupervised graph-based clustering of the integrated dataset identified 21 transcriptionally distinct clusters (Figure 2). Major cell populations were assigned based on canonical marker gene expression and the top differentially expressed genes per cluster. Immune cells constituted the largest fraction of recovered cells and were dominated by T-cell populations, characterised by expression of *CD3D/CD3E* and cytotoxic markers such as *NKG7*, *PRF1*, and granzymes, indicating the presence of multiple CD8^+^ T-cell states. A smaller lymphoid compartment corresponding to B cells was identified by expression of MS4A1 (*CD20*), *CD74*, and *BANK1*.

The myeloid compartment included monocyte/macrophage subsets, supported by expression of *LYZ*, antigen presentation-related genes (e.g., *HLA-DMA*), and subtype-specific markers such as *CD14/FCN1* (classical monocytes) and *CD36/TREM2* (macrophage-like cells). Neutrophil clusters were distinguished by granulocyte-associated markers (e.g., *GPR84*, *CXCR1*, *S100A8/S100A9*) and also included an additional cluster with a stress-response signature characterised by increased expression of heat-shock and stress-related transcripts. A mast cell cluster was detected based on *KIT* expression.

Epithelial populations comprised luminal epithelial cells with a strong expression of milk protein genes (*CSN1S1*, *CSN2*, *CSN3*, *LALBA*) and associated secretory markers, as well as luminal progenitor-like cells showing increased expression of epithelial junction/keratin-related genes (e.g., *KRTs*, *CLDNs*, *WFDC2*). A cycling cluster was identified by high expression of cell cycle genes (*MKI67*, *TOP2A*, *STMN1*) and was consistent with proliferating immune cells.

### 3.5. Deeply Sequenced Clusters

**Figure 3:**
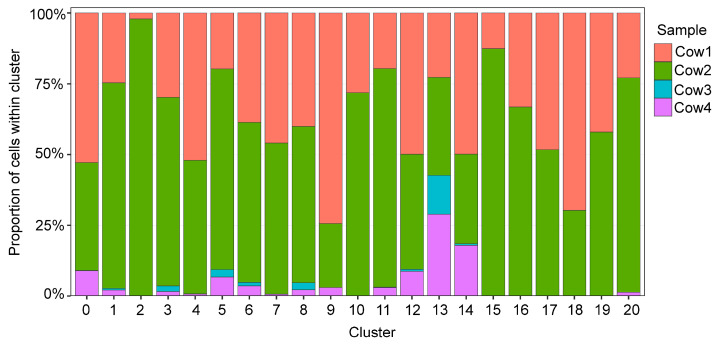
Cluster composition by biological sample.

Clusters enriched for cells derived from Cow3 and Cow4, which were sequenced at substantially higher depth, exhibited increased transcriptomic complexity and clearer separation of functional subpopulations. These deeply sequenced clusters included several immune populations, most notably CD8^+^ T-cell clusters (clusters 0, 1, 3, 5, 8, 9, 11, 12, 17, and 19), cycling T cells (cluster 20), and multiple monocyte subtypes (clusters 4, 7, and 15). In addition, luminal epithelial clusters associated with milk protein expression (clusters 13, 16, and 18) were strongly enriched in deeply sequenced samples (Figure 3). In contrast, clusters dominated by cells from the previously published datasets, which contained a higher number of cells but lower sequencing depth per cell, primarily represented broader cell classes such as neutrophils and monocytes. These clusters showed reduced transcriptional resolution and fewer detectable low-abundance transcripts.

### 3.6. Transcription Factor Expression and Sequencing Depth Effects

To characterise transcriptional regulatory activity in bovine milk somatic cells, we analyzed the expression of transcription factors (TFs) across the integrated scRNA-seq dataset. Transcription factors were identified based on a curated list obtained from AnimalTFDB, and their expression levels were summarized across all cells following normalization and data integration.

**Figure 4:**
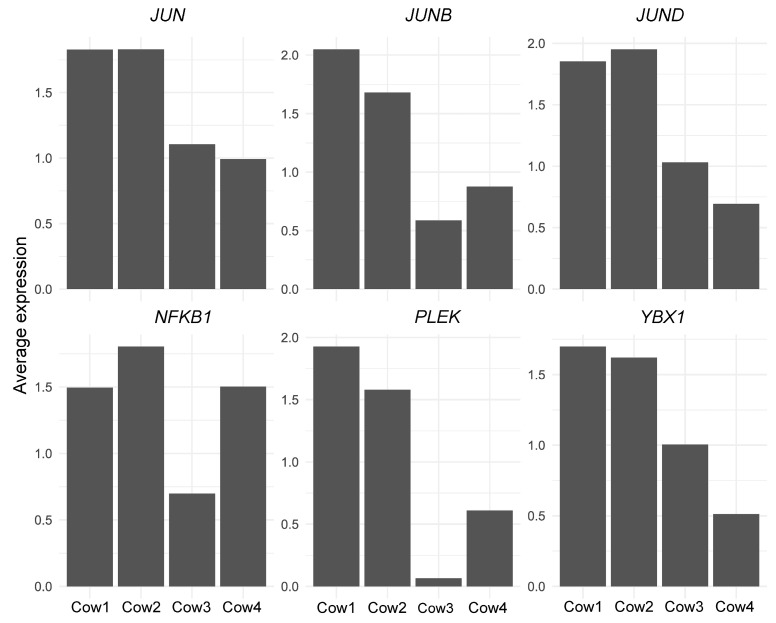
Detected expression of transcription factors. across bovine milk somatic cell scRNA-seq datasets. Bar plots show the average normalized expression of the transcription factors *JUN*, *JUNB*, *JUND*, *NFKB1*, *PLEK*, and *YBX1* in samples (Cow1–Cow4).

When ranked according to their average expression across the entire dataset, the most highly expressed transcription factors included *JUN*, *JUNB*, *JUND*, *NFKB1*, *PLEK*, and *YBX1* (Figure 4). These TFs are known regulators of immune activation, stress response, and epithelial function, and their high expression is consistent with the cellular composition of bovine milk, which is dominated by immune and secretory epithelial cells. The consistent detection of these TFs across all datasets indicates that they represent core transcriptional programmes active in milk-derived cells.

To assess the impact of sequencing depth on transcription factor detection, TF expression profiles were further compared between deeply sequenced samples (Cow3 and Cow4) and datasets with lower sequencing depth (Cow1 and Cow2). While the majority of highly expressed TFs were detected in all datasets, deeper sequencing enabled the detection of additional low-abundance transcription factors that were not reliably observed in shallow datasets.

Notably, *NANOG* was detected exclusively in the deeply sequenced samples. This transcription factor was absent or below the detection threshold in datasets with lower read depth, indicating that its detection was dependent on increased transcriptome coverage rather than differences in cell composition. The low-level expression of *NANOG* suggests the presence of rare transcriptional states associated with cellular plasticity rather than a distinct stem cell population. Its detection highlights the sensitivity gain achieved through deeper sequencing and illustrates how sequencing depth influences the ability to resolve subtle regulatory features in milk-derived cells.

Overall, these results demonstrate that while shallow sequencing is sufficient for identifying major cell populations and broadly expressed transcription factors, increased sequencing depth improves detection of low-abundance regulatory genes and provides a more detailed view of transcriptional heterogeneity in bovine milk somatic cells.

## 4. Discussion

In this study, we performed an integrated single-cell transcriptomic analysis of bovine milk somatic cells, combining newly generated high-depth scRNA-seq data with previously published datasets [17]. By applying a uniform analytical pipeline and integrating datasets with different sequencing depths, we provide a comprehensive view of cellular heterogeneity in bovine milk and highlight how sequencing depth influences the resolution of transcriptional programmes.

Consistent with previous studies [16,17,18], the major cellular compartments identified in bovine milk included immune cells and epithelial populations. T lymphocytes constituted the dominant immune fraction, with multiple CD8^+^ T-cell subpopulations characterised by expression of cytotoxic markers such as *NKG7*, *PRF1*, and granzymes. Monocytes, macrophages, neutrophils, mast cells, and B cells were also detected, confirming the complex immune composition of milk reported previously in cattle [16,17,18] and other mammals [14,27]. Epithelial populations were represented by luminal epithelial cells expressing canonical milk protein genes (*CSN1S1*, *CSN2*, *CSN3*, *LALBA*) and by luminal progenitor–like cells marked by keratins and tight junction–associated genes. These findings further support the concept that milk-derived cells faithfully reflect the cellular and functional diversity of the lactating mammary gland.

A key observation of this study is that, although overall cell-type composition was largely consistent across datasets, sequencing depth strongly influenced the resolution of transcriptional states within these populations. Datasets generated with higher sequencing depth showed increased transcriptome complexity per cell and enabled the detection of lowly expressed genes that were not reliably captured in shallower datasets. This effect was particularly evident within immune and epithelial populations, where deeper sequencing revealed additional transcriptional heterogeneity without altering overall cluster identity.

Analysis of transcription factor expression further illustrated this effect. Across the integrated dataset, the most highly expressed transcription factors included *JUN*, *JUNB*, *JUND*, *NFKB1*, *PLEK*, and *YBX1*, all of which are known regulators of immune activation, cellular stress response, and epithelial function. *JUN* and its paralogs *JUNB* and *JUND* are functional components of the *AP-1* transcription factor complex and enhance its transcriptional activity. *NFKB1* is a pleiotropic transcription factor present in many cell types and is the endpoint of a series of signal transduction events. *PLEK* and *YBX1* are involved in G protein-coupled receptor signalling pathway and regulation of transcription, pre-mRNA splicing and translation, respectively. These factors were consistently detected across samples and likely reflect core transcriptional programmes active in milk-derived immune and epithelial cells.

Comparison between deeply sequenced and lower-depth datasets revealed that certain transcription factors were detectable only when sequencing depth was sufficient. Among these, *NANOG* was identified exclusively in the deeply sequenced samples. Although expressed at low levels and in a limited subset of cells, its detection is biologically noteworthy. *NANOG* is a key regulator of cellular plasticity and stem-like transcriptional programmes [28,29], and its presence in milk-derived cells may reflect transient progenitor-like or plastic cell states within the mammary epithelium. The absence of *NANOG* signal in lower-depth datasets suggests that such regulatory programmes may remain undetected without sufficient transcriptome coverage, rather than being truly absent.

The detection of *NANOG* in this study does not imply the presence of classical pluripotent stem cells in milk. Rather, it likely reflects low-level expression associated with epithelial plasticity or stress-responsive transcriptional states, which have been reported in mammary tissue during lactation and remodeling [30]. Consistent with this interpretation, low-frequency *NANOG* expression in milk-derived epithelial cells has also been reported in mature-stage human breast milk cells [31]. This observation supports the view that milk somatic cells represent a dynamic population with transcriptional features extending beyond terminal differentiation.

These findings emphasize that sequencing depth influences not only quantitative metrics such as gene counts but also the qualitative interpretation of regulatory programmes. While shallow datasets are sufficient for identifying major cell types and estimating their proportions, deeper sequencing enables detection of subtle transcriptional states, low-abundance regulators, and potential plasticity-related signals that would otherwise remain obscured.

Despite providing high-resolution single-cell transcriptomic insights into bovine milk somatic cells, this study has several limitations. The number of animals included in the newly generated datasets was limited to two cows, which restricts the ability to generalize quantitative differences in cell-type proportions and transcriptional states across the broader dairy population. In addition, samples were collected at a single stage of lactation and therefore do not capture the dynamic transcriptional changes that occur across early, peak, and late lactation. The lactation stage is known to strongly influence mammary gland physiology, immune activity, and epithelial remodeling, and future studies should incorporate longitudinal sampling across multiple lactation phases. Additionally, collecting and analyzing milk from separate quarters could provide better insight in cellular dynamics in different parts of the mammary gland.

Future research should aim to expand both the biological and comparative scope of milk-derived single-cell analyses. Increasing the number of animals and integrating samples from multiple lactation stages will allow a more robust assessment of inter-individual variability and temporal transcriptional dynamics. In addition to transcriptomic profiling, the integration of single-cell chromatin accessibility approaches, such as single-cell ATAC-seq, would enable direct investigation of regulatory landscapes underlying transcriptional plasticity and low-abundance regulatory programmes identified in this study. Extending these integrative single-cell approaches to other dairy species, such as goat and sheep, will further enable cross-species comparisons of mammary epithelial and immune cell states, helping to identify conserved and species-specific regulatory mechanisms associated with lactation. Such comparative analyses may provide valuable insights into the evolution of mammary gland function and support the development of translational frameworks linking milk cell biology across species.

In conclusion, our study provides an integrated single-cell transcriptomic reference of bovine milk somatic cells and demonstrates that sequencing depth plays a crucial role in resolving transcriptional complexity within this system. The identification of low-abundance transcription factors such as *NANOG* highlights the value of high-resolution transcriptomic profiling for uncovering regulatory features associated with cellular plasticity in the lactating mammary gland. These findings contribute to a more refined understanding of mammary gland biology and provide a framework for future studies investigating functional heterogeneity, lactation biology, and health-related traits in dairy cattle.

## Figures and Tables

**Table 1:** Summary of sequencing metrics for bovine milk somatic cell scRNA-seq datasets.

Metric	Cow1	Cow2	Cow3	Cow4
Number of cells	10,073	17,781	263	1386
Mean reads per cell	35,883	12,011	1,300,409	286,841
Median genes per cell	1029	953	1126	382
Median UMI * counts per cell	2341	1702	4774	970
Sequencing saturation (%)	77.2	63.2	94.2	94.8
Fraction reads in cells (%)	90.3	86.7	21.2	27.0
Valid barcodes (%)	98.7	97.9	82.8	92.2
Valid UMIs (%)	100.0	99.9	99.8	99.9
Reads mapped to genome (%)	96.6	94.5	87.5	87.1
Reads mapped confidently to genome (%)	93.7	91.4	82.7	84.5
Reads mapped confidently to exonic regions (%)	77.0	65.8	64.7	55.8
Total genes detected	26,126	29,023	19,547	22,655

* UMI—unique molecular identifier.

**Table 2:** Summary of cell filtering and doublet removal across bovine milk somatic cell scRNA-seq datasets.

Dataset	Cells Before QC *	Cells Removed During QC	Doublets Removed	Cells Retained
Cow1	10,073	389	763	8921
Cow2	17,781	455	1420	15,906
Cow3	263	12	11	240
Cow4	1386	325	69	992

* QC—quality control.

## Data Availability

The datasets generated and/or analyzed during the current study are available in the European Nucleotide Archive (ENA) repository, under accession number PRJEB73560. Previously published samples are referred to as Cow1 (sample accession SAMEA115396859) and Cow2 (SAMEA115396860). Newly generated high-depth datasets are referred to as Cow3 (SAMEA120903505) and Cow4 (SAMEA120903506).
